# Factors associated with overweight/obesity of children aged 6–12 years in Indonesia

**DOI:** 10.1186/s12887-023-04321-6

**Published:** 2023-09-25

**Authors:** Sofi Oktaviani, Mayumi Mizutani, Ritsuko Nishide, Susumu Tanimura

**Affiliations:** 1 Department of Public Health Nursing, Mie University Graduate School of Medicine, 2-174, Edobashi, Tsu, Mie 514-8507 Japan; 2 Indramayu College of Health Science, Indramayu, Indonesia

**Keywords:** Child, Indonesia, Obesity, Overweight, Pediatric obesity

## Abstract

**Background:**

Globally, the prevalence of childhood obesity has increased considerably, including in Indonesia. Obesity results from multifactorial interactions at the personal, familial, and environmental levels. However, little is known about the factors associated with overweight/obesity among children in Indonesia. This study is intended to identify personal, familial, and environmental factors associated with overweight/obesity in children aged 6–12 years in Indonesia.

**Methods:**

Study design was a secondary data analysis using the Indonesia Family Life Survey in 2014/2015, focusing on 6,090 children aged 6–12 years. The questions covered the child’s body mass index and potential personal, familial, and environmental factors. Logistic regression analysis was performed to identify the personal, familial, and environmental factors.

**Results:**

The mean age of participants was 8.9 years (SD = 2.0); 51.0% were boys; 9.4% were overweight; and 8.1% were obese. Overweight and obesity were associated with age [AOR 1.09 (95% CI 1.04–1.14)], having an overweight [AOR 1.93 (95% CI 1.58–2.36)] or obese [AOR 3.36 (95% CI 2.43–4.61)] father compared with a normal father, being of Chinese [AOR 9.51 (95% CI 1.43–79.43)] or Javanese [AOR 1.60 (95% CI 1.16–2.24)] ethnicity compared with Sundanese ethnicity, and residing in an urban area [AOR 1.36 (95% CI 1.10–1.70)]. A lower risk of child overweight/obesity was associated with the father’s perception [AOR 0.56 (95% CI 0.38–0.80)] and mother’s perception [AOR 0.66 (95% CI 0.43–0.98)] of the child’s food consumption as being less than adequate compared with adequate.

**Conclusions:**

Risk factors in children for overweight/obesity were older age, having an overweight/obese father, membership of certain ethnic groups, and urban residence. The main protective factor was parents’ perception that a child’s food consumption was less than adequate. Health promotion programs focused on these factors could help control or prevent childhood obesity in Indonesia.

## Background

Overweight and obesity among children are becoming a crucial public health issues in lower-middle-income countries (LMICs) such as Indonesia, which have lagged high-income countries (HICs) where overweight and obesity began to increase significantly from as early as the mid-1980s [1, 2]. Globally, the prevalence of overweight and obesity among children and adolescents aged 5–19 years has approximately doubled between 1996 and 2016, from 8.9 to 18.4%, respectively, and it has tripled in LMICs, from 3.8 to 11.2%. In Indonesia, the prevalence of overweight and obesity among children and adolescents aged 5–19 years increased fourfold, from 3.9 to 15.4%, between 1996 and 2016, respectively [3]. Meanwhile, in 2018, 10.8% and 9.2% of children aged 5 − 12 years were overweight and obese, respectively [4].

Overweight and obesity during childhood and adolescence can result in short-term adverse consequences including high-blood pressure, [5–8] obstructive sleep apnea, [9] and severe COVID-19, [10–12] as well as long-term consequences, including adult obesity [13] and higher mortality risk: children with obesity were at three times greater risk of premature death than normal children [14]. Overweight and obesity are not only caused by personal characteristics but they also reflect multifactorial interactions of personal, familial, environmental, and cultural factors [15].

Among personal factors, overweight and obesity has been associated with high consumption of obesogenic food, for instance, fast food, snacks, ultra-processed food, and sweet beverages; [16, 17] sedentary behavior; [18] and sleep time [19]. Family-level factors include education, [20, 21] parents’ nutritional status, [17, 19, 22] and parents’ food consumption [23]. A systematic review and meta-analysis found residence in rural or urban areas to be an environmental-level factor contributing to children being overweight and obese, [24, 25] and a 2018 qualitative study identified the diverse ways in which culture influences food preferences that potentially contribute to overweight and obesity [26].

However, the above-referenced literature has few gaps that need to be clarified in future studies. For instance, while some studies have focused entirely on how mothers influence children’s nutritional status [17], little attention has been paid to how fathers influence children’s nutritional status. Moreover, weight and height data from Indonesian studies are based on self-reporting from parents, and these data might differ from direct measurement results [19]. Moreover, inconsistencies in research findings related to the relative impacts of rural and urban residence on overweight and obesity in HICs and LMICs [24, 25] need to be resolved. Lastly, although previous researchers have investigated environment-level impacts of culture on food preferences, [26] few have identified associations between cultural factors and children’s nutritional status. Due to the high level of cultural diversity in Indonesia, future studies should aim to clarify the relationship between cultural diversity and children’s nutritional status in the country.

This study focused on children aged 6–12 years. An ecological study among 34 provinces in Indonesia found that children aged 5–12 years had a higher prevalence of overweight/obesity than adolescents (aged 13–15 and 16–18 years) [27]. In addition, body mass index (BMI) changes during childhood, and children’s BMI begins to increase after six years [28, 29]. The present study was intended to fill these research gaps by identifying personal, familial, and environmental factors associated with overweight and obesity among children aged 6–12 years in Indonesia, an LMIC.

## Methods

### Survey design and study population

Study design was a secondary data analysis using data from the fifth wave of the Indonesia Family Life Survey (IFLS-5), an extension to 2014/2015 of an ongoing longitudinal survey that was conducted jointly by the RAND Corporation in the United States and University Gadjah Mada in Indonesia. IFLS-5 based on a sample of household represented approximately 83% of the Indonesian population living in 13 of the country’s 27 provinces in 1993. Provinces were selected to represent Indonesia’s population and to capture its cultural diversity. From each province, 321 enumeration areas were randomly chosen from the nationally representative sample frame used in the 1993 National Socioeconomic Survey. Twenty households were randomly selected from each urban enumeration area, and 30 were randomly selected from each rural enumeration area. In the subsequent survey waves, the original household and split-off household were recontacted. IFLS-5 included 16,931 households, a 28.2% overlap with the total of 60,000 households that participated in the 1993 National Socioeconomic Survey [30].

To be included in the data analysis for this study, participants had to be children aged 6–12 years old and their parents, for whom data on weight and height were available to calculate BMI. To focus on either children of normal weight or overweight children, as our primary exclusion criterion, we excluded thin or underweight children (BMI-for-age z-score (BAZ) < − 2SD); we also excluded children who did not live with their parents. The total number of children aged 6 − 12 years, as detected in IFLS-5, was 8780. After we filtered out all duplicated data (n = 455), missing data on the child’s weight and height (n = 1135), children classified as thin or underweight (n = 632), and children who did not live with their parents (n = 468), we had data available for analysis from 6,090 children.

### Survey questions

**Fig. 1:**
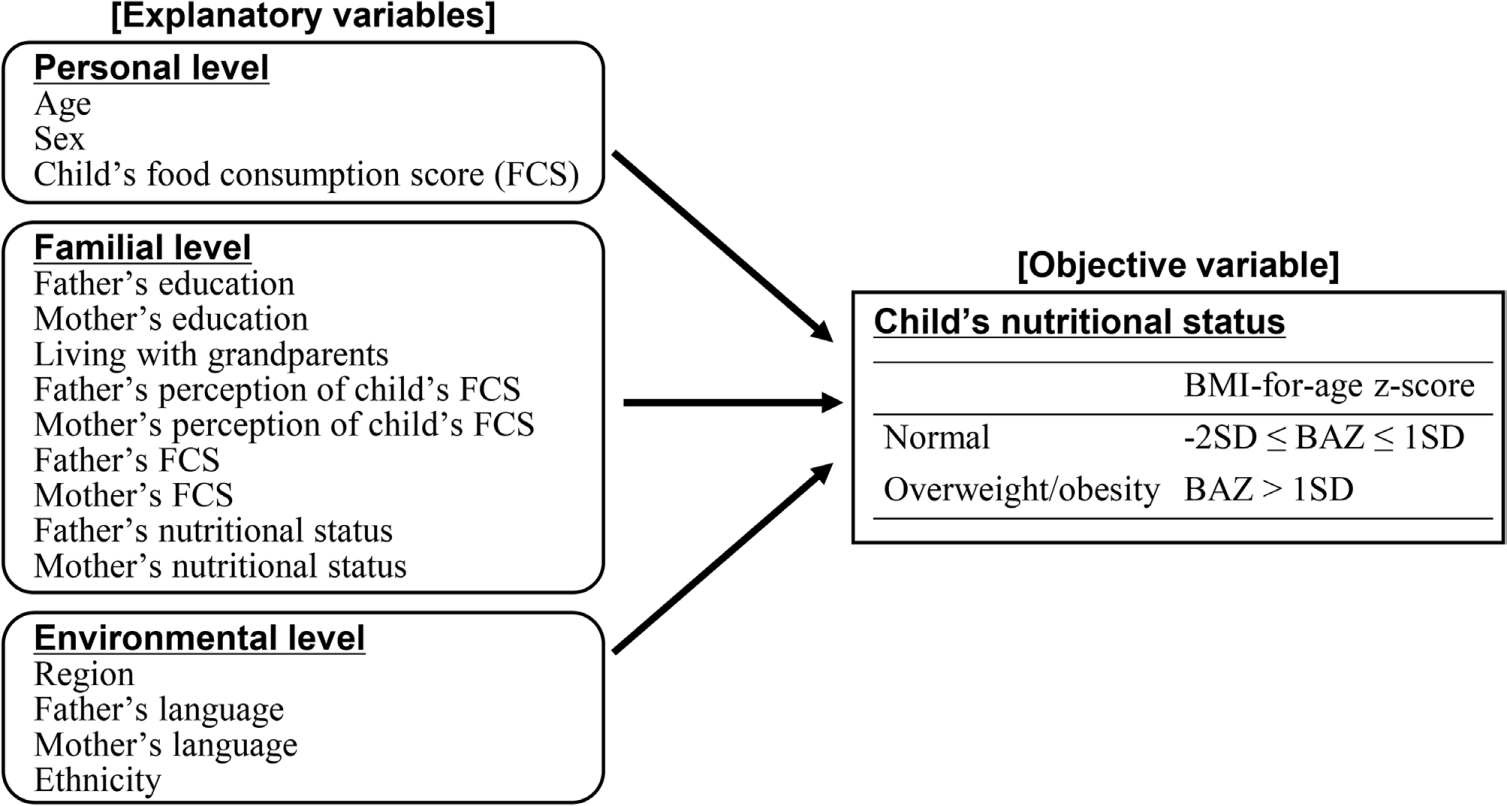
Conceptual framework for nutritional status and potential influencing factors among Indonesian children aged 6–12

This study used a framework for understanding obesity in children and youth, [31] which explains that changes in individual characteristics are a result of multifactorial interactions, including personal factors (e.g., age, gender, and genetic profile), behavioral settings (e.g., home and school), and the environmental contexts in which people live. We focus on some variables from this framework that are available in a questionnaire from IFLS-5. We used survey questions to capture potential personal, familial, and environmental factors that could contribute to overweight and obesity in children. Figure 1 shows the conceptual framework of this study.

The personal-level potential factors were the children’s age, sex, and food consumption score (FCS). The World Food Programme defines the FCS as “a score calculated using the frequency of consumption of different food groups consumed by a household during the 7 days before the survey,” noting “there are standard weights for each of the food groups that comprise the FCS” [32]. IFLS-5 documented consumption of 11 food items (leafy green vegetables, carrots, bananas, papayas, mangos, sweet potatoes, rice, meat, fish, eggs, and dairy) categorized into five groups: vegetables (green leafy vegetables and carrots), fruit (bananas, papayas, and mangos), staples (sweet potatoes and rice), protein (meat, fish, and eggs), and dairy. We categorized food consumption based on the FCS as poor (< 21), borderline (21–35), or acceptable (> 35) [33].

The family-level potential factors were the parents’ education, whether children lived with or without their grandparents, parents’ perceptions of their children’s food consumption as well as the parents’ FCS, and parents’ nutritional status. For education level, the questionnaire included a question on the highest level of education attained by the parents, and we grouped their responses into one of five categories: no school, primary school, middle school, high school, or higher education. There were four options in the question for parent’s perceptions of their children’s food consumption: “it is less than adequate for their needs,” “it is just adequate for their needs,” “it is more than adequate for their needs,” and “do not know.” We categorized parents’ FCS according to the World Food Programme scoring, and we classified parents’ BMI (body weight in kilograms divided by the square of body height in meters) as underweight (BMI < 18.5), normal (18.5 ≤ BMI < 25), overweight (25 ≤ BMI < 30), or obese (BMI ≥ 30) following the guidelines of the World Health Organization (WHO) [34].

For the potential environmental factors, we looked at the region and cultural diversity factors such as ethnicity and language. For region, we used the IFLS-5 question that asks whether respondents live in an urban or a rural area. Indonesia has approximately 1,300 ethnicities, [35] and IFLS-5 included a multiple-choice list for parents to choose from; for this study, we focused on the following 26 ethnicities: Sundanese, Acehnese, Ambon, Bali, Banjar, Banten, Batak, Betawi, Bima-Dompu, Bugis, Cirebon, Chinese, Dayak, Javanese, Komering, Maduranese, Makasar, Manado, Melayu, Minang, Nias, Palembang, Sasak, Sumbawa, Other Southern Sumatrans, and Toraja. We classified parents’ languages as Indonesian, other than Indonesian, or Indonesian and other languages. The category “other than Indonesian” includes local languages that participants used.

Weight and height were measured by the trained interviewers of IFLS-5. Interviewers learned how to take physical health measurements during training. Heights were measured using a Seca plastic height board, model 213, which measured children’s height to the nearest millimeter. Weights were measured using a Camry model EB1003 scale, which measured children’s weight to the nearest tenth of a kilogram [30]. We calculated the child’s BAZ using a method approved by the WHO and classified it as normal (− 2SD ≤ BAZ ≤ 1SD) or overweight/obese (BAZ > 1SD) [36]. In this study, we used the WHO 2007 R macro package to calculate children’s BAZ [37].

### Statistical analysis

We conducted data analysis using the following steps. First, we calculated descriptive statistics for all variables. Then, we conducted bivariate analysis using t test and Fisher’s exact test to identify relationships between objective and explanatory variables, excluding variables with perfect separation (i.e., outcome variable separates a predictor variable completely) from the multivariate analyses. We deleted missing values listwise. Univariate and multivariate analysis was conducted by specifying logistic regression models. Crude and adjusted odds ratios (ORs and AORs) were calculated for each variable. We also computed adjusted generalized variance inflation factors (GVIFs) to detect potential multicollinearity in the models [38]. We set significance at p < 0.05 for the t test and Fisher’s exact test, and for the logistic regression models, we set significance at a 95% confidence interval (CI). We analyzed the data using R version 4.0.5 [39].

## Results

Data gathered from 6,090 children aged 6–12 years that met inclusion criteria were analyzed. Table 1 shows the results of the descriptive and bivariate analysis of the children’s nutritional status and potential factors. The mean age was 8.9 years (SD = 2.0) (not presented in the table), and the sex ratio was 104. More than half of the participants lived in urban areas (59.5%). One-fifth of fathers (21.9%) and mothers (21.5%) spoke both Indonesian and other languages. The ethnic group with the highest proportion was Javanese (39.9%), followed by Sundanese (12.2%), Minang (6.1%), and Batak (6.0%). The percentages of overweight and obese children were 9.4% and 8.1%, respectively. Half of the mothers were overweight/obese (50.1%), while one-third of the fathers were overweight/obese (30.8%). In two-thirds of cases, the child’s FCS was acceptable (68.5%), but fewer fathers (61.6%) and mothers (54.8%) perceived that their child’s food consumption was just adequate for their needs.

**Table 1:** Participants’ characteristics and bivariate analysis of child’s nutritional status and potential factors

			Child’s nutritional status				*P-value*
	Total (n = 6090)		Normal (n = 5022)		Overweight/ obesity (n = 1068)		
	n	%	n	%	n	%	
**Personal level**							
Age (n = 6090)							
6 years old	921	15.1	773	83.9	148	16.1	0.134
7 years old	869	14.3	734	84.5	135	15.5	
8 years old	918	15.1	768	83.7	150	16.3	
9 years old	900	14.8	732	81.3	168	18.7	
10 years old	858	14.1	698	81.4	160	18.6	
11 years old	891	14.6	732	82.2	159	17.8	
12 years old	733	12.0	585	79.8	148	20.2	
Sex (n = 6090)							
Boys	3105	51.0	2535	81.6	570	18.4	0.890
Girls	2985	49.0	2487	83.3	498	16.7	
Child’s FCS (n = 6079)							
Acceptable (> 35)	4166	68.5	3405	81.7	761	18.3	0.022
Borderline (21–35)	1720	28.3	1439	83.7	281	16.3	
Poor (< 21)	193	3.2	170	88.1	23	11.9	
**Familial level**							
Father’s education (n = 5452)							
No school	107	2.0	101	94.4	6	5.6	< 0.001
Primary school	1703	31.2	1507	88.5	196	11.5	
Middle school	1056	19.3	900	85.2	156	14.8	
High school	1896	34.8	1513	79.8	383	20.2	
Higher education	690	12.7	492	71.3	198	28.7	
Mother’s education (n = 5831)							
No school	120	2.0	109	90.8	11	9.2	< 0.001
Primary school	1814	31.1	1585	87.4	229	12.6	
Middle school	1322	22.7	1141	86.3	181	13.7	
High school	1835	31.5	1439	78.4	396	21.6	
Higher education	740	12.7	532	71.9	208	28.1	
Living with grandparents (n = 6081)							
Yes	1375	22.6	1121	81.5	254	18.5	0.294
No	4706	77.4	3895	82.8	811	17.2	
Father’s perception of child’s food consumption (n = 4593)							
Less than adequate	679	14.8	635	93.5	44	6.5	< 0.001
Just adequate	2831	61.6	2356	83.2	475	16.8	
More than adequate	1083	23.6	848	78.3	235	21.7	
Mother’s perception of child’s food consumption (n = 5554)							
Less than adequate	648	11.7	587	90.6	61	9.4	< 0.001
Just adequate	3046	54.8	2563	84.1	483	15.9	
More than adequate	1860	33.5	1456	78.3	404	21.7	
Father’s FCS (n = 4639)							
Acceptable (> 35)	2884	62.2	2388	82.8	496	17.2	0.112
Borderline (21–35)	1586	34.2	1338	84.4	248	15.6	
Poor (< 21)	169	3.6	149	88.2	20	11.8	
Mother’s FCS (n = 5589)							
Acceptable (> 35)	3354	60.0	2765	82.4	589	17.6	0.121
Borderline (21–35)	2037	36.5	1693	83.1	344	16.9	
Poor (< 21)	198	3.5	174	87.9	24	12.1	
Father’s nutritional status (n = 4758)							
Underweight	344	7.2	322	93.6	22	6.4	< 0.001
Normal	2949	62.0	2577	87.4	372	12.6	
Overweight	1202	25.3	909	75.6	293	24.4	
Obesity	263	5.5	167	63.5	96	36.5	
Mother’s nutritional status (n = 5657)							
Underweight	207	3.7	167	80.7	40	19.3	0.011
Normal	2621	46.3	2209	84.3	412	15.7	
Overweight	1932	34.2	1582	81.9	350	18.1	
Obesity	897	15.9	716	79.8	181	20.2	
**Environmental level**							
Region (n = 6090)							
Rural	2467	40.5	2170	88.0	297	12.0	< 0.001
Urban	3623	59.5	2852	78.7	771	21.3	
Father’s language (n = 5051)							
Indonesia	733	14.5	558	76.1	175	23.9	< 0.001
Other	3211	63.6	2765	86.1	446	13.9	
Indonesia and other	1107	21.9	881	79.6	226	20.4	
Mother’s language (n = 5711)							
Indonesia	888	15.5	666	75.0	222	25.0	< 0.001
Other	3600	63.0	3060	85.0	540	15.0	
Indonesia and other	1223	21.5	1002	81.9	221	18.1	
Ethnicity (n = 6056)							
Sundanese	740	12.2	617	83.4	123	16.6	< 0.001
Acehnese	11	0.2	5	45.5	6	54.5	
Ambon	2	0.0	2	100.0	0	0.0	
Bali	286	4.7	236	82.5	50	17.5	
Banjar	203	3.4	165	81.3	38	18.7	
Banten	24	0.4	24	100.0	0	0.0	
Batak	362	6.0	315	87.0	47	13.0	
Betawi	283	4.7	214	75.6	69	24.4	
Bima-Dompu	118	1.9	109	92.4	9	7.6	
Bugis	259	4.3	221	85.3	38	14.7	
Cirebon	2	0.0	0	0.0	2	100.0	
Chinese	14	0.2	8	57.1	6	42.9	
Dayak	3	0.0	2	66.7	1	33.3	
Javanese	2415	39.9	1919	79.5	496	20.5	
Komering	19	0.3	17	89.5	2	10.5	
Maduranese	135	2.2	117	86.7	18	13.3	
Makasar	123	2.0	109	88.6	14	11.4	
Manado	2	0.0	1	50.0	1	50.0	
Melayu	39	0.6	28	71.8	11	28.2	
Minang	368	6.1	305	82.9	63	17.1	
Nias	40	0.7	39	97.5	1	2.5	
Palembang	60	1.0	47	78.3	13	21.7	
Sasak	253	4.2	234	92.5	19	7.5	
Sumbawa	25	0.4	23	92.0	2	8.0	
Other Southern Sumatrans	234	3.9	203	86.8	31	13.2	
Toraja	36	0.6	33	91.7	3	8.3	

Fisher’s exact test. FCS: food consumption score

From the results of the bivariate analysis using t test (mean age) and Fisher’s exact test, 12 of 16 potential factors were related to the child’s nutritional status. The mean age of overweight/obese children was significantly higher than that of children of normal weight (9.1 vs. 8.9, p < 0.001) (not presented in the table). More overweight/obese children lived in urban areas than in rural areas (21.3% vs. 12.0%, p < 0.001). A higher prevalence of childhood overweight/obesity was associated with a higher educational level of the father (28.7%, p < 0.001) and mother (28.1%, p < 0.001). The prevalence was higher if the father (21.7%, p < 0.001) and mother (21.7%, p < 0.001) perceived their child as consuming more than an adequate amount of food. The higher prevalence was associated with an overweight (24.4%) and obese father (36.5%, p < 0.001), but it was also seen in underweight mothers (19.3%, p = 0.011). The prevalence was higher if language of the father (23.9%, p < 0.001) and mother (25.0%, p < 0.001) was the Indonesian language. Some ethnicities were prone to higher prevalence such as Acehnese with 54.5% (p < 0.001).

Table 2 shows the results of the logistic regression models. A univariate logistic regression revealed an association between the child’s nutritional status and 13 factors: age, child’s FCS, father’s and mother’s education, father’s and mother’s perception of the child’s food consumption, mother’s FCS, father’s and mother’s nutritional status, father’s and mother’s language, ethnicity, and region. A multivariate logistic regression revealed the association between the child’s nutritional status and six factors: age, father’s perception of the child’s food consumption, mother’s perception of the child’s food consumption, father’s nutritional status, ethnicity, and region. A higher risk of child overweight/obesity was associated with older age which increases age by each one year increased the odds being overweight or obese by 9% (AOR = 1.09, 95% CI: 1.04–1.14), an overweight father (AOR = 1.93, 95% CI: 1.58–2.36) or obese father (AOR = 3.36, 95% CI: 2.43–4.61), Chinese (AOR = 9.51, 95% CI: 1.43–79.43) or Javanese ethnicity (AOR = 1.60, 95% CI: 1.16–2.24), and residing in an urban area (AOR = 1.36, 95% CI: 1.10–1.70). In contrast, a lower risk of child overweight/obesity was associated with the father (AOR = 0.56, 95% CI: 0.38–0.80) and mother (AOR = 0.66, 95% CI: 0.43–0.98) perceiving their child’s food consumption as being less than adequate. The GVIF ranged from 1.01 to 1.23, indicating no multicollinearity between the explanatory variables.

**Table 2:** Logistic regression model identifying factors associated with overweight/obesity among children

	Unadjusted			*P-value*	Adjusted				*P-value*
	OR	95% CI			OR	95% CI			
**Personal level**									
Age^a^	1.06	1.01	1.10	0.014	1.09	1.04	1.14	< 0.001	
Sex									
Boys	1.00	(ref)			1.00	(ref)			
Girls	0.89	0.75	1.06	0.191	0.94	0.79	1.13	0.537	
Child’s FCS									
Acceptable	1.00	(ref)			1.00	(ref)			
Borderline	0.82	0.67	0.99	0.042	0.97	0.78	1.22	0.813	
Poor	0.63	0.33	1.12	0.138	1.00	0.49	1.88	0.992	
**Familial level**									
Father’s education									
No school	1.00	(ref)			1.00	(ref)			
Primary school	2.83	1.03	11.66	0.081	2.41	0.82	10.33	0.159	
Middle school	3.16	1.14	13.10	0.055	2.23	0.74	9.69	0.205	
High school	5.84	2.15	24.00	0.003	3.06	1.03	13.26	0.076	
Higher education	8.43	3.07	34.85	< 0.001	3.45	1.12	15.17	0.054	
Mother’s education									
No school	1.00	(ref)			1.00	(ref)			
Primary school	1.10	0.55	2.53	0.802	0.65	0.30	1.58	0.303	
Middle school	1.26	0.62	2.90	0.553	0.61	0.28	1.51	0.254	
High school	2.22	1.12	5.07	0.035	0.81	0.36	2.02	0.634	
Higher education	2.85	1.41	6.58	0.007	0.76	0.33	1.94	0.534	
Living with grandparents									
No	1.00	(ref)			1.00	(ref)			
Yes	1.16	0.93	1.43	0.177	1.10	0.87	1.38	0.438	
Father’s perception of child’s food consumption									
Less than adequate	0.36	0.25	0.50	< 0.001	0.56	0.38	0.80	0.002	
Just adequate	1.00	(ref)			1.00	(ref)			
More than adequate	1.38	1.14	1.67	< 0.001	1.00	0.81	1.24	0.976	
Mother’s perception of child’s food consumption									
Less than adequate	0.40	0.27	0.58	< 0.001	0.66	0.43	0.98	0.043	
Just adequate	1.00	(ref)			1.00	(ref)			
More than adequate	1.56	1.30	1.86	< 0.001	1.17	0.96	1.43	0.128	
Father’s FCS									
Acceptable	1.00	(ref)			1.00	(ref)			
Borderline	0.88	0.73	1.06	0.184	0.99	0.81	1.22	0.938	
Poor	0.64	0.36	1.06	0.099	1.04	0.57	1.80	0.890	
Mother’s FCS									
Acceptable	1.00	(ref)			1.00	(ref)			
Borderline	0.93	0.77	1.11	0.398	1.01	0.82	1.25	0.892	
Poor	0.48	0.24	0.85	0.020	0.80	0.38	1.50	0.506	
Father’s nutritional status									
Underweight	0.36	0.20	0.62	< 0.001	0.41	0.22	0.70	0.002	
Normal	1.00	(ref)			1.00	(ref)			
Overweight	2.38	1.97	2.87	< 0.001	1.93	1.58	2.36	< 0.001	
Obesity	4.09	3.01	5.53	< 0.001	3.36	2.43	4.61	< 0.001	
Mother’s nutritional status									
Underweight	1.39	0.88	2.12	0.142	1.31	0.81	2.07	0.257	
Normal	1.00	(ref)			1.00	(ref)			
Overweight	1.24	1.02	1.50	0.032	1.15	0.94	1.41	0.180	
Obesity	1.37	1.07	1.74	0.012	1.21	0.93	1.57	0.149	
**Environmental level**									
Region									
Rural	1.00	(ref)			1.00	(ref)			
Urban	2.17	1.80	2.62	< 0.001	1.36	1.10	1.70	0.005	
Father’s language									
Indonesia	1.00	(ref)			1.00	(ref)			
Other	0.54	0.43	0.69	< 0.001	0.84	0.60	1.18	0.317	
Indonesia and other	0.83	0.64	1.09	0.176	0.99	0.72	1.35	0.941	
Mother’s language									
Indonesia	1.00	(ref)			1.00	(ref)			
Other	0.53	0.42	0.66	< 0.001	0.83	0.60	1.16	0.280	
Indonesia and other	0.64	0.49	0.83	<0.001	0.75	0.54	1.03	0.074	
Ethnicity									
Sundanese	1.00	(ref)			1.00	(ref)			
Aceh	6.76	0.80	57.31	0.058	5.67	0.63	52.11	0.100	
Bali	1.55	0.98	2.44	0.057	1.40	0.85	2.26	0.179	
Banjar	1.68	1.01	2.73	0.040	1.70	0.99	2.88	0.051	
Batak	0.73	0.43	1.20	0.226	0.70	0.40	1.19	0.198	
Betawi	2.57	1.65	4.00	< 0.001	1.62	1.00	2.61	0.048	
Bima-dompu	0.27	0.06	0.75	0.029	0.41	0.10	1.21	0.156	
Bugis	1.02	0.59	1.71	0.944	1.20	0.67	2.09	0.527	
Chinese	10.15	1.65	78.32	0.012	9.51	1.43	79.43	0.020	
Javanese	1.64	1.21	2.26	0.001	1.60	1.16	2.24	0.004	
Komering	1.04	0.16	3.90	0.958	1.36	0.20	5.37	0.696	
Maduranese	1.14	0.54	2.23	0.704	1.33	0.60	2.73	0.462	
Makasar	0.80	0.36	1.62	0.560	0.85	0.37	1.79	0.682	
Melayu	1.42	0.40	3.96	0.534	1.24	0.34	3.64	0.711	
Minang	1.37	0.86	2.14	0.175	1.12	0.69	1.80	0.650	
Nias	0.25	0.01	1.21	0.178	0.66	0.04	3.41	0.689	
Palembang	1.87	0.76	4.12	0.141	1.81	0.69	4.30	0.198	
Sasak	0.59	0.31	1.06	0.090	0.63	0.32	1.17	0.161	
Sumbawa	0.34	0.02	1.67	0.294	0.35	0.02	1.86	0.321	
Other Southern Sumatrans	0.87	0.50	1.46	0.598	1.09	0.61	1.90	0.755	
Toraja	1.27	0.29	3.96	0.712	1.14	0.25	3.83	0.850	

^a^ Numerical data. Outcome variable: nutritional status of overweight/obese child (normal-weight child = reference). OR: odds ratio, CI: confidence interval

## Discussion

This study revealed a childhood overweight/obesity rate of 17.5% among Indonesian children aged 6–12 years. This prevalence has increased in Indonesia since 2007 (12.8%) [17]. The increasing trend indicates a need to address and control Indonesia’s rate of overweight and obesity among children.

One personal factor we identified as being associated with a higher risk of childhood overweight and obesity was the child’s age. This study used BAZ to classify the child’s nutritional status. Although we adjusted BMI by age, it was associated with overweight and obesity, possibly because, as they age, children make more independent decisions [40]. In Indonesia, primary school students have a high exposure to less nutritious foods, [4, 17, 41] and are less physically active, [4] and it is challenging for children who have only begun to develop their own decision-making skills to make good food choices. Thus, high exposure to less nutritious food with less physical activity increases the likelihood that children will consume these foods, leading them to become overweight and obese.

Family factors that were associated with childhood overweight and obesity were having an overweight or obese father and parents’ perceptions of their children’s food consumption. In this study, children of overweight or obese fathers were two to four times more likely to be overweight or obese themselves, consistent with a systematic review and meta-analysis from HICs, middle-income countries, and one low-income country in which child obesity was associated with overweight or obesity among fathers [22]. The elevated risk is likely attributable to the combination of genetic predisposition and shared environmental factors. However, according to social learning theory, parents’ actions directly influence their children’s behaviors through experience and observations, [42] and some children likely imitate their parents’ obesity-promoting behaviors.

Whereas a significant association exists between paternal and childhood overweight/obesity, no such association has been found between maternal and childhood overweight/obesity. A qualitative study conducted in Indonesia [43] revealed that fathers have described themselves as more permissive, whereas mothers tend to be more overprotective. Meanwhile, a nine-year prospective cohort study found that authoritative parenting was perceived as more successful at preventing children from increased BMI than permissive parenting [44]. According to social learning theory, [42] children imitate each other’s behavior through a process known as reproduction. Indonesian fathers tend to have more permissive parenting styles, and they also tend to indulge their children by giving them everything they need. This may give children more opportunity to replicate their fathers’ obesity-promoting behavior in the reproduction process. In addition, Javanese fathers are expected to be imitation models for their children, [45] which made fathers the main models for the children’s behavior. This mechanism might explain why different studies have reached different results concerning the association between parental nutritional status and overweight/obesity among children in Indonesia.

We also found that parents who perceived their children as having less than adequate food consumption tended to have children with normal weight. Studies have demonstrated unique cultural perceptions; for example, Indonesian adults often found overweight children to be “cute[r],” “health[ier],” and “funn[ier]” [46, 47]. These culturally held beliefs may contribute to childhood overweight/obesity, as overweight children may be more appealing. Additionally, significant familial variation exists in the definition of a healthy diet [48].

We also found some environmental factors to be associated with childhood overweight/obesity, specifically, living in an urban area and being of Chinese or Javanese ethnicity. Understanding cultural differences in eating habits could elucidate this finding. Apart from providing sustenance, food plays a social and cultural role by establishing and maintaining interpersonal relationships. For example, Chinese mothers in China often use sweets and desserts as rewards for their children, [49] and excessive consumption of sweet foods potentially contributes to the increased overweight and obesity that we observed among Chinese children in Indonesia. Similarly, a nationwide health survey in Indonesia found that Javanese people (who live in Central Java province, East Java province, and the Special Region of DI Yogyakarta) consumed more sweets per day than did Sundanese people (who live in West Java province) [50]. Indeed, the cuisine of Central Java, where 70% of the inhabitants are ethnic Javanese, tends to be very sweet [51]. Meanwhile, findings from a cross-sectional study conducted in England corroborated possible associations between childhood overweight and obesity and ethnic and cultural factors [52].

We also found that children living in urban areas were 1.36 times more likely to be overweight or obese than were children living in rural areas, and this finding was consistent with findings from studies conducted in Indonesia and China [53, 54]. Urban areas are considered obesogenic environments with high access to less nutritious foods [25]. Additionally, data from the Indonesian National Health Survey revealed that people from urban environments more often had sedentary lifestyles than did people from rural environments [50].

Results of this study will afford better understanding of children, familial, and environmental characteristics in Indonesia, which public health nurses can use to provide health promotion and intervention programs for those who suffer from nutritional problems. Moreover, our study found that fathers play an important role in influencing overweight and obesity among children in Indonesia. While health education was traditionally provided only for women and children in Indonesia, future prevention strategies to overcome overweight and obesity among children must also include fathers. The implication of this study is that urban areas could become targeted areas for future intervention or prevention. However, Indonesia has regional disparities that lead to huge gaps in socioeconomic development between western Indonesia and central/eastern Indonesia. For a more targeted approach, future studies also need to capture socioeconomic differences at the regional level that might also contribute to childhood overweight and obesity.

This study is the first of its kind to provide data on personal, familial, and environmental factors associated with childhood overweight and obesity in Indonesia using national data. This study has several potential limitations. First, it used self-reported questionnaire data from IFLS, which may introduce bias. Questions in the survey addressing parents’ perceptions about their children’s food consumption may be biased. Parents were asked to subjectively qualify their children’s eating habits as less than adequate, just adequate, or more than adequate. It is unclear how parents who participated in this questionnaire define each category on perception about children’s food consumption that could lead to misclassification. Second, we were not able to determine causal relationships between childhood overweight and obesity and the factors we studied because of the cross-sectional study design. Third, although one ethnicity showed an association with childhood overweight/obesity, this association was present only in a small sample; thus, the result may not be replicated in other studies. Fourth, we were unable to include some potentially confounding variables (e.g., family income, physical activity, and sugar-beverage consumption) because they were either inconsistent or unavailable in the IFLS-5 data. Fifth, we did not incorporate sample weight in the analysis, which means that this study cannot clearly explain the extent to which its results represent the total Indonesian population. Although the baseline sample in the IFLS-1 represents 83% of the Indonesian population, decreasing the recontact rate of original households in 1993 [30] could lead to a decrease in representativeness. In addition, we included in the multivariate analysis only participants with complete data. Some characteristics significantly differed between data included in and excluded from the analysis (data not shown). Finally, differences in participants’ characteristics potentially led to selection bias.

## Conclusions

Among children aged 6–12 years in Indonesia, overweight/obesity was associated with the following personal, familial, and environmental risk factors: age, overweight or obese father, ethnicity (i.e., Chinese and Javanese), and living in an urban area. Normal childhood weight was associated with parents’ perceptions that children’s food consumption was less than adequate. Targeting the different factors we identified as significant on multiple levels could be a critical first step in increasing community-wide insight and improving nursing approaches to preventing primary childhood overweight and obesity.

## Data Availability

The datasets are available upon registration on the website of the RAND Corporation (https://www.rand.org/well-being/social-and-behavioral-policy/data/FLS/IFLS.html).

## Abbreviations

BMI: body mass index
IFLS: Indonesia Family Life Survey
FCS: food consumption score
WHO: World Health Organization
OR: odds ratio
AOR: adjusted odds ratio
GVIF: generalized variance inflation factor
CI: confidence interval
